# A sustainable bio-based char as emerging electrode material for energy storage applications

**DOI:** 10.1038/s41598-024-51350-x

**Published:** 2024-01-11

**Authors:** Gabriela Hristea, Mihai Iordoc, Eduard-Marius Lungulescu, Iuliana Bejenari, Irina Volf

**Affiliations:** 1 National Research and Development Institute for Electrical Engineering ICPE-CA, 313 Splaiul Unirii, Bucuresti, Romania; 2 Faculty of Chemical Engineering and Environmental Protection, Gheorghe Asachi Technical University of Iasi, 73 Prof. D. Mangeron Street, 700050 Iasi, Romania

**Keywords:** Environmental sciences, Energy science and technology, Engineering

## Abstract

In the last few years, extensive research efforts have been made to develop novel bio-char-based electrodes using different strategies starting from a variety of biomass precursors as well as applying different thermochemical conversion paths. In this regard, hydrothermal carbonization method is becoming a more prevalent option among conversion procedures even if pyrolysis remains crucial in converting biomass into carbonaceous materials. The main aim of this study is to develop an innovative supercapacitor electrode from spruce bark waste through a unique low-temperature technique approach, which proved to effectively eliminate the pyrolysis step. Consequently, a hybrid spruce-bark-graphene oxide compound (HySB) was obtained as electrode material for supercapacitors. When compared to a regularly used commercial electrode material, SLC1512P graphite (reference) with 150.3 µF cm^−2^ capacitance, the HySB has a substantially higher capacitive performance of 530.5 µF cm^−2^. In contrast to the reference, the HySB polarization resistance increases by two orders of magnitude at the stationary potential and by three orders of magnitude at the optimum potential, underlying that the superior performances of HySB extend beyond static conditions. The synthesis strategy provides an appropriate energy-efficient option for converting biomass into carbonaceous materials with meaningful properties suitable for energy storage applications.

## Introduction

Biomass resources (vegetable, farming, and animal wastes, organic wastes, and industrial byproducts) have a high water and oxygen content and poor calorific value which have a detrimental impact on disposal quality. Physical treatments (size reduction-crushing and grinding, drying, filtration, aggregation) and different methods of conversion (biochemical and thermochemical processes) can reduce these drawbacks and lead to an upper-level processing^1,2^.

The solid phase (char) generated through thermochemical conversion of biomass has the potential to be processed and refined for numerous applications including, wastewater treatment^3^, catalysis^4^, natural carrier and recently within the energy conversion and storage area^5^. In this regard, electrode designs based on biomass-derived particles are used in supercapacitor (SC) applications^6–13^.

A supercapacitor (SC) (also called an electrochemical capacitor) is an energy storage system that can supply high energy in a short period of time by working reversibly. In these devices, electrode architecture, composition, and surface properties are crucial for improving energy output. Electric double-layer capacitance (EDLCs) and pseudocapacitance (PC) are mainly involved in charge mechanisms of storage in supercapacitors^8,11^.

It has been thoroughly investigated how materials with primarily PC behaviour as well as those with EDLC characteristics can be employed to develop efficient electrodes for SCs^10^. Carbons, particularly natural graphite, which are often used as electrode materials^14,15^ are critical components in the production of SCs. As well, carbons are the most adaptable material in converting and storing energy^16,17^ due to its desirable chemical and physical properties such as tunable porous structure, large surface area, excellent electrical conductivity, and good chemical/thermal stability. However, the most frequent carbon materials used in supercapacitors, such as activated carbons, carbon nanotubes and graphene-like materials limit their broad application due to high manufacturing costs and laborious preparation processes.

Recognizing that biomass is a renewable, cost-effective and environmentally friendly resource, processing biomass-based nano/microparticles for electrochemical applications has gained increased interest. Several carbon-based materials, including activated carbon, biochar, or hybrids can be formed from various biomass precursors^18–22^. For instance, hierarchical porous biochars (HPBCs) such biochar carbon tubes (BCTs), biochar carbon fibres (BCFs) and biochar graphene (BG), have been modelled and functionalized to meet different needs of electrode materials in supercapacitors^23^. Through carbonization and activation of biomass precursors, Yang et al. demonstrated a clear template-free production approach to produce hierarchical porous HPBCs^24^. The resulting biochar exhibits an electrochemical capacitance of 587 F g^−1^ in an aqueous electrolyte. Also, sulphur, nitrogen, phosphorus, or calcium, which are inherently integrated heteroatoms in the chars structure, could provide a key function in electrode materials as electron donors, influencing the surface's hydrophilicity, electron conductivity and cycling stability^25–27^. The inclusion of hydrophilic functional groups associated with heteroatoms can enhance also the ability of biochar-based electrodes to interact with and be wetted by liquids^28^. The advantages of especially oxygen–nitrogen–sulphur co-doped bio-based chars for applications as supercapacitor electrodes have been reported^29–31^. Multi-heteroatom-doped carbon displayed a greater specific capacitance compared with a mono-heteroatom-doped material^30^.

Regardless the significant accomplishments in environmentally friendly operations research, bio-based char applications in energy storage field remain limited since the variability of precursors contributes to chars’ functional performance. Therefore, in order to meet more requirements from a sustainable view as well as from energy storage perspective, new research directions should be opened up to develop feasible and predictable bio-based char composite materials (BCMs).

This study’s breakthroughs are primarily based on: (1) to design and develop a bio-based char SCs electrode with an active mass-loading of around 10 mg cm^−2^ and a thickness of 5 µm, (2) to innovate the preparation approach in order to use less energy-intensive methods in the processing path and (3) to highlight the superior performance of the new product SCs electrode compared to a commercial carbon electrode used for the same application.

To the best of our knowledge only a few studies have been reported the use of spruce bark-based char in the development of an electrode for supercapacitors^32–34^ none of them using a low-temperature conversion approach.

The main challenge of this research was not to improve the porosity of the electrode material, which already arises from the inherent porosity of spruce bark, but to enhance the electrical conductivity by directly attaching graphene materials to the material surface without any intermediary steps, in order to enhance its electrical conductivity, improve electrochemical stability, and the wetting capability of the electrode. Wetting degree and electrochemical characteristics were emphasized as the main critical factors affecting electrode performance.

## Results and discussion

### Morphology of the samples

**Figure 1:**
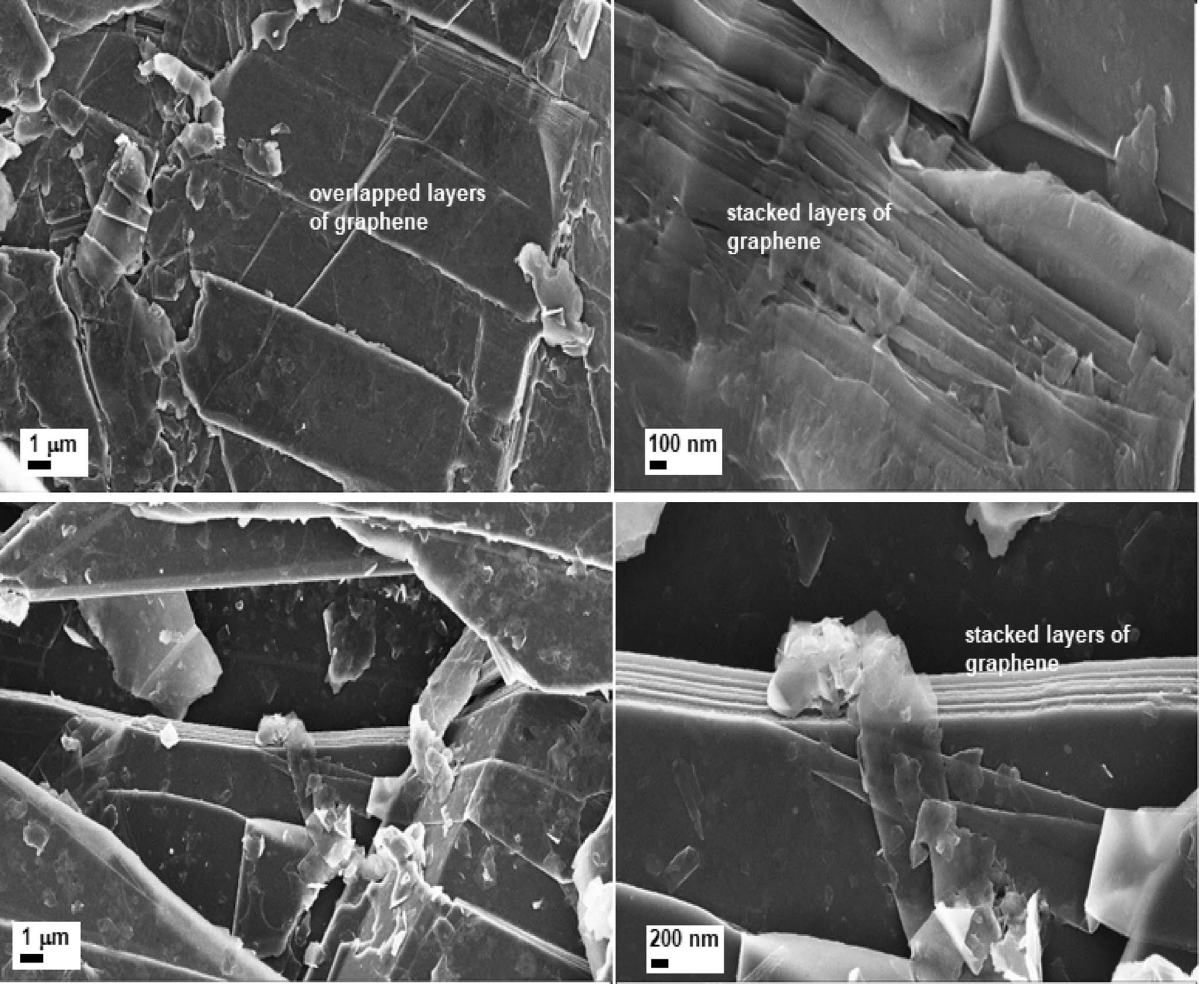
Scanning electron microscopy images of graphite oxide (GO)-layered structure. The electron microscopy images highlight the morphology of the analyzed samples. Graphite oxide has a multilayered structure that is close in appearance to graphite. Knowing that graphite is mainly composed of numerous layers of graphene, in Fig. 1, the stratified structure formed by these multiple layers becomes clearly observable.

**Figure 2:**
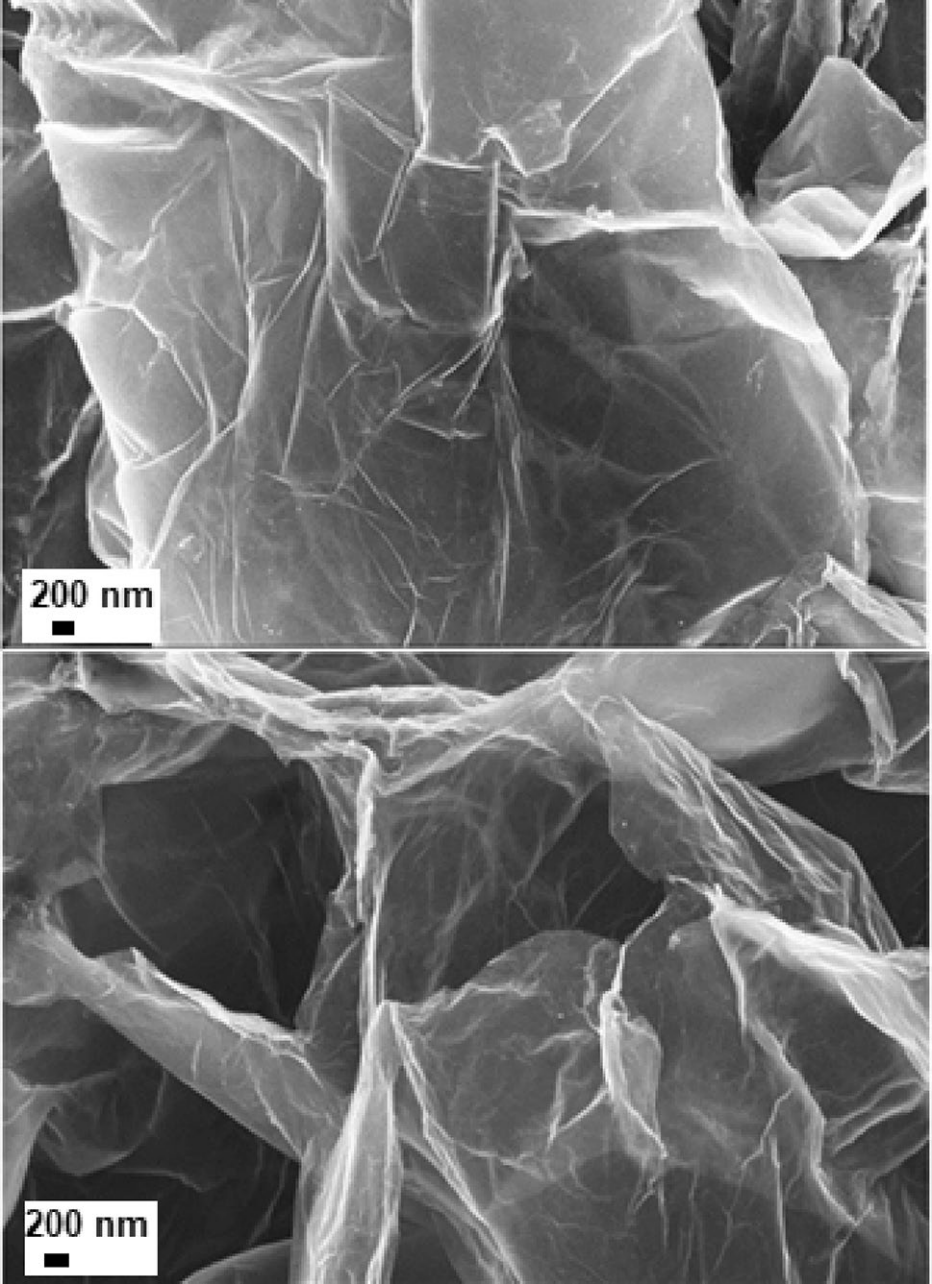
Scanning electron microscopy images of graphene oxide (GnO)—expanded graphene sheets. The number of layers distinguishes graphite oxide from graphene oxide. The scanning electron microscopies images in Fig. 2. show that the stack layers of graphene become thinner, and the spacing of the graphene layers expanded compared with the stack layered structure shown in Fig. 1.

Figure 1 shows the graphite oxide morphology exhibiting a stacked structure and random wrinkled layer structure. Graphite oxide has a layered structure similar to that of graphite. The important morphological changes in GO upon sonication became clearly visible in Fig. 2. This indicates an aerated porous structure, showing an expanded matrix with larger spaces between the multi-layer graphene sheets. By sonication graphene-stacked layers from graphite oxide (GO), are largely exfoliated. The cavitation-induced pressure pulsations are responsible for the evolution and collapse of microbubbles in liquids during sonication. Cavitation produces shock waves, which generate shear stresses on the carbon substrate. The sonic waves, that cause the solvent molecules to penetrate between the layers, leads to the transformation of multilayer GO into less-layers form.

As mentioned earlier, Fig. 1 illustrates the graphite oxide structure in a dense plane stacking state. After sonication treatment, the stack layers of graphene become thinner, and the layers spacing expanded as in Fig. 2.

Even though there are other additional techniques that can be applied, including, exfoliation in supercritical fluids, ball milling, etc. the yields of graphene produced by these alternate methods are often modest^35–38^. In this context, exfoliation and dispersion by sonication remain a highly efficient alternative, fast, and cost-effective for industrially synthesizing and reducing of graphene oxide.

**Figure 3:**
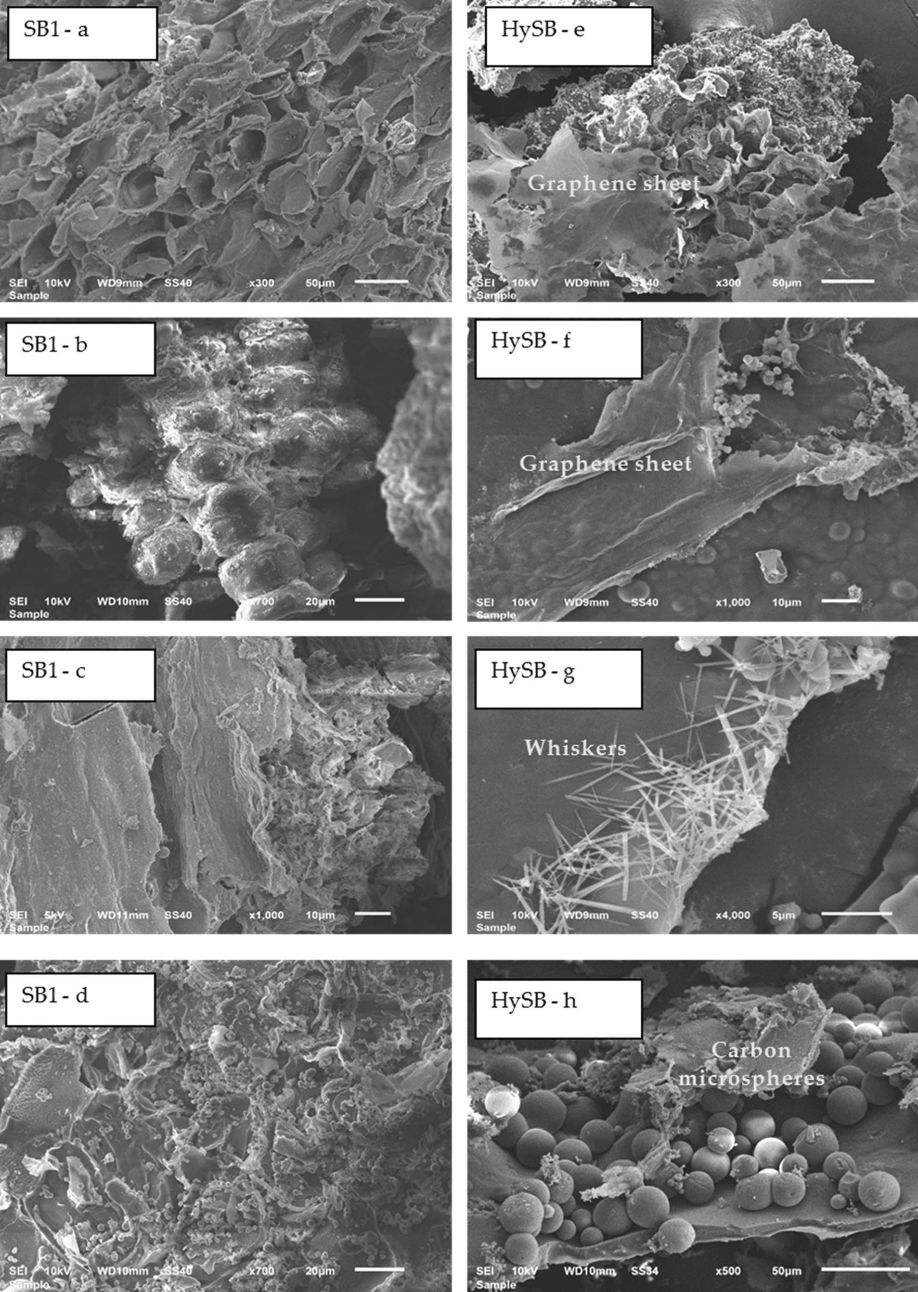
Scanning electron microscopy images of SB1 sample (**a**–**d**) compared with morphologies of HySB sample (**e**–**h**). SEM images put in evidence distinct morphological features of the hydrochar SB1 and HySB hybrid compound. **e** and **f** make clear the availability of graphene sheets, as well as the large abundance of carbon microspheres (shown in **h**) and whiskers structures (**g**). In the case of the SB1 sample, carbon microspheres are also detectable (**d**), but they appear to be substantially smaller in size.

Figure 3 shows electron microscopy images of the SB bio-based char sample (Fig. 3a-d) in contrast to the HySB (Fig. 3e-h). The HySB sample appears to develop a higher diversity of morphologies than the precursor.

The presence of graphene sheets, as seen in Fig. 3e,f, as well as a high abundance of carbon microspheres (shown in Fig. 3h) along with the presence of whiskers structures, is clearly evident. Carbon microspheres are also visible in the case of the SB1 sample (Fig. 3d), but they appear to be significantly smaller in size. The formation of such carbon microsphere structures has been reported in different studies^39^ as a consequence of low-temperature hydrothermal synthesis^40,41^.

**Figure 4:**
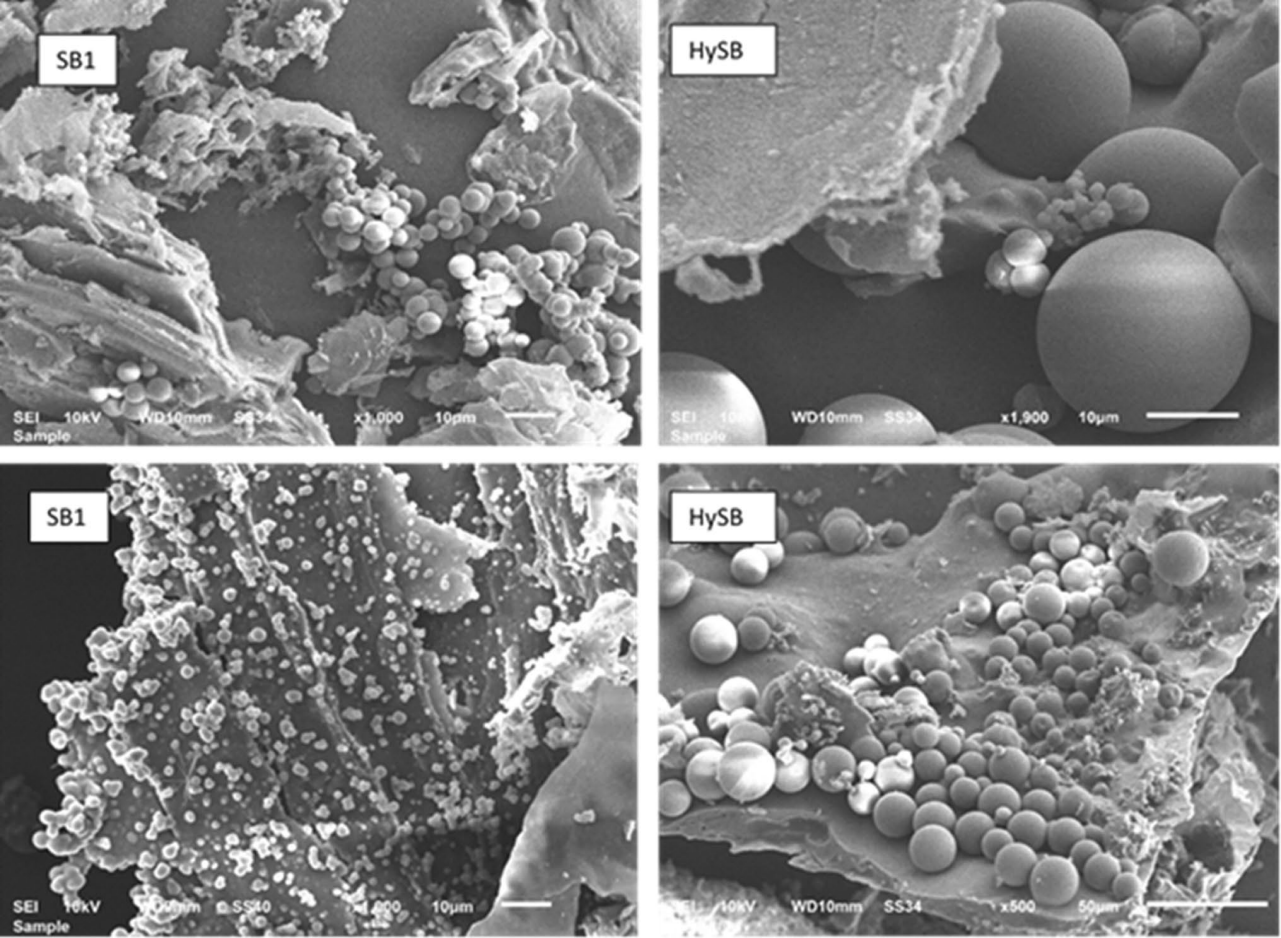
Scanning electron microscopy images of carbon microspheres in SB1 and HySB samples. The electron microscopy images emphasize the structural characteristics of the SB1 and HySB hydro chars, revealing a significant contrast in size between the developed carbon microspheres.

Figure 4 depicts the considerable variation in size between the SB1 and HySB carbon microsphere samples.

### Raman analysis

Raman spectroscopy is a powerful technique used to analyse the vibrational modes of materials by measuring their scattering of light, providing valuable insights into the structural properties and quality of carbon-based materials (e.g., graphene oxide, reduced graphene oxide). By analysing the characteristic peaks in the Raman spectra, it can be assessed the degree of oxidation, the presence of defects, and the level of reduction in these materials^42^.

**Figure 5:**
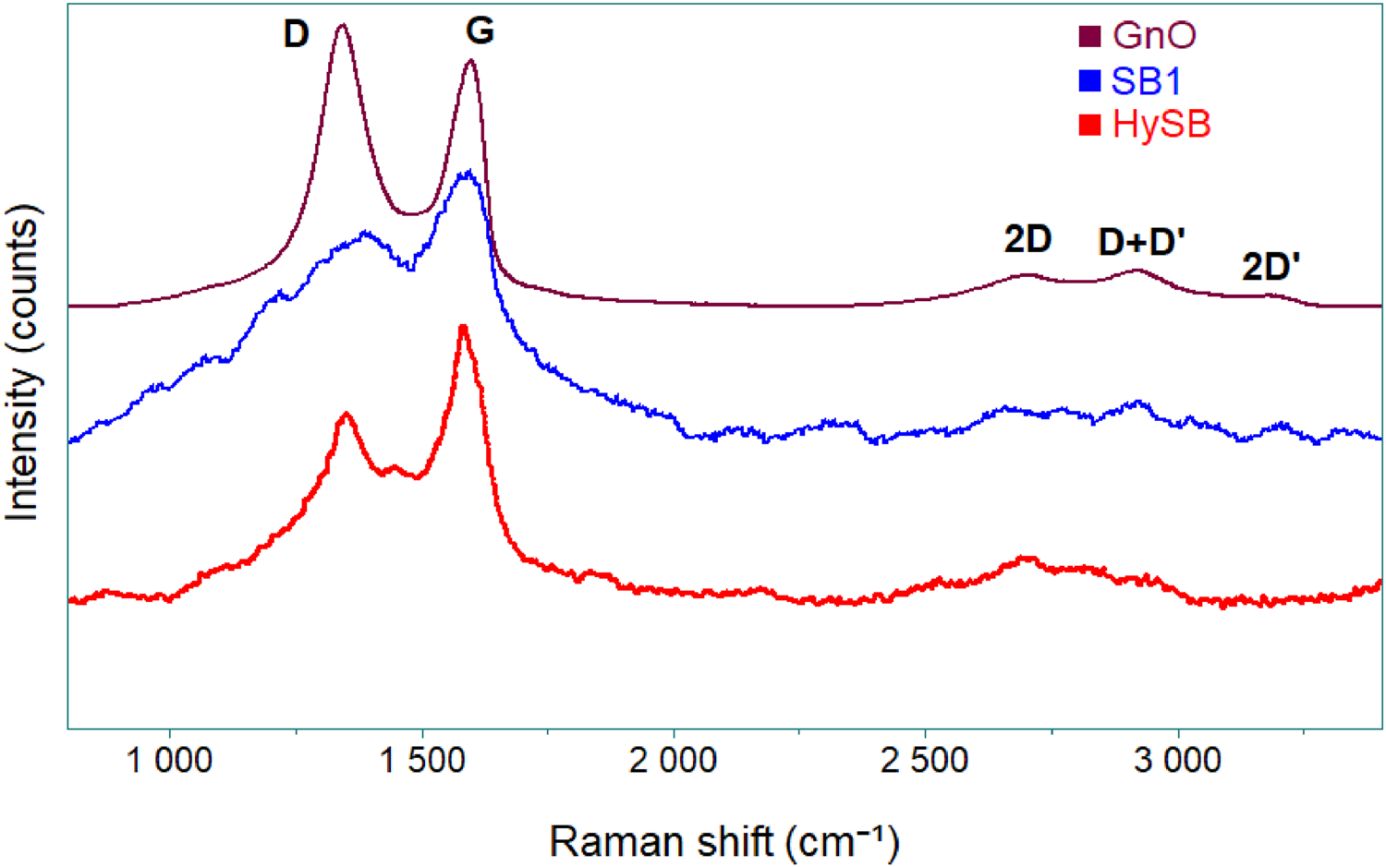
Raman spectra recorded on analyzed samples. The general Raman spectrum profile (peak position and relative peak intensity) provides a unique chemical fingerprint that can be used to identify a material and distinguish it from others.

The Raman spectra recorded on the analysed samples (i.e., GnO, SB1, and HySB) are presented in Fig. 5. All Raman spectra exhibit characteristic bands (Table 1) of carbonaceous structures^42–45^: (1) D band (between 1349 and 1389 cm^−1^ which arises from the presence of structural defects and disorder in the carbon lattice); (2) G band (between 1582 and 1593 cm^−1^ which arises from the in-plane vibrational motion of sp2 carbon atoms in the graphene lattice); (3) 2D band (between 2600–2900 cm^−1^, which corresponds to the second-order Raman scattering process and provides information about the number of graphene layers and the stacking arrangement).

**Table 1:** Raman bands characteristics to the analyzed samples.

Sample	D position, cm^−1^	G position, cm^−1^	I_D_/I_G_
GnO	1342	1593	1.15
SB1	1389	1589	0.77
HySB	1349	1582	0.68

The Raman spectrum recorded for the GnO sample (Fig. 5) exhibits similarities to those reported in the literature for graphene oxide^43^, thus confirming the successful formation of graphene oxide through the exfoliation of graphite flakes. Thus, the GnO sample is characterized by a relatively high ID/IG ratio (1.15), indicating an increased degree of disorder and a greater prevalence of defects in the graphene oxide structure^46^. These observations can be attributed to the presence of oxygen functional groups and the introduction of structural defects during the oxidation process.

After undergoing the first cycle of hydrothermal carbonization, the structure of spruce bark transforms into a carbonaceous structure, as evident from the Raman spectrum (Fig. 5). The spectrum exhibits a prominent D band centred at approximately 1389 cm^−1^ and a G band at 1589 cm^−1^. The presence of a weak 2D band suggests a low degree of stacking in the material^46^. Upon subjecting the sample to a second hydrothermal cycle, in combination with graphene oxide (GnO) (Fig. 5), a more well-defined D band is observed, with a red-shifted position at 1349 cm^−1^. The G band is centred around 1582 cm^−1^, and the intensity of the 2D band has increased. This alteration is indicative of the enhanced structural quality and a more graphene-like nature in the obtained material, resembling reduced graphene oxide. The decrease in the ID/IG ratio (Table 1) from 1.15 (GnO) to 0.77 (first cycle) and further to 0.68 (second cycle) supports this observation. It suggests a reduction in the number of defects, an increase in the graphitic characteristics, and a restoration of the sp2 carbon bonding network (as indicated by the increased intensity of the G band)^45^. Due to this fact, the HySB material possesses enhanced electrical properties, resulting in efficient charge transfer, making it an excellent candidate for electrode applications, as shown in the electrochemical evaluation section.

### FTIR analysis

FTIR spectroscopy (Fourier Transform Infrared spectroscopy) is a technique used to analyse the functional groups and chemical bonds present in a material, providing valuable information about their structural and chemical properties.

**Figure 6:**
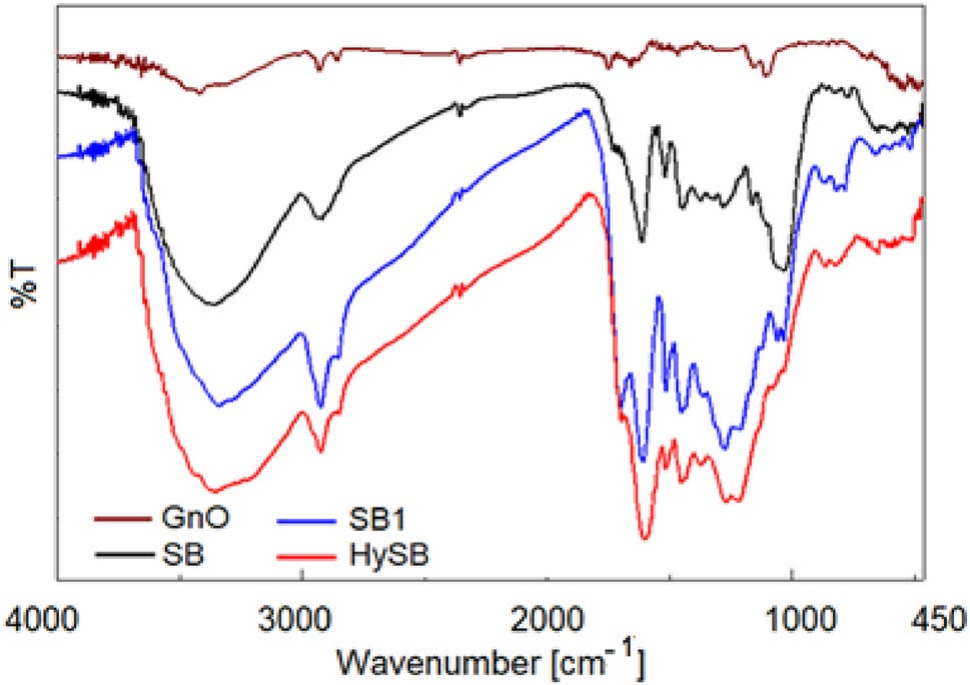
FTIR spectra recorded on analysed samples. Fourier Transform Infrared Spectroscopy (FTIR) identifies chemical bonds/ functional groups in a molecule by producing an infrared absorption spectrum. The spectra produce a profile of the sample, a distinctive molecular fingerprint.

FTIR spectroscopy analysis of graphene oxide (Fig. 6) has confirmed the presence of various oxygen-containing functional groups^45,47^. The spectra have revealed the existence of hydroxyl groups, indicated by a broad peak observed between 3100 and 3600 cm^−1^, carbonyl groups detected within the range of 1700–1800 cm^−1^, while carboxyl groups appear between 1600 and 1700 cm^−1^. The presence of C=C bonds is evidenced by peaks observed in the 1500−1600 cm^−1^ range, and ether or epoxide groups are detected between 1000 and 1200 cm^−1^. Also, peaks related to C-H stretching vibrations (both symmetric and asymmetric) can be observed in the 2820–2980 cm^−1^ range^48^.

The FTIR spectrum of neat spruce bark (Fig. 6) present characteristic bands of hemicellulose, cellulose, lignin and polysaccharides^49^: 3100–3680 cm^−1^ (–OH stretching), 2800–3000 cm^−1^ (symmetric and asymmetric C–H stretching), 1600–1750 cm^−1^ (various C=O bond types stretch vibrations), 1500–1540 cm^−1^ (aromatic C=C stretching vibrations), 1400–1480 cm^−1^ (C=C and C–H bond), 1184–1380 cm^−1^ (C–H, C=O and C–O vibrations) and 900–1084 cm^−1^ (C–O stretch). The application of the first hydrothermal cycle results in a slight modification of the SB spectrum, primarily characterized by an increased intensity of the peak at 1695 cm^−1^ (C=O from carboxyl), accompanied by a decrease in intensity in the 900–1084 cm^−1^ region (ester C–O–C bond) and 1084–1380 cm^−1^. Furthermore, this peak shows a split into two distinct peaks at 1063 cm^−1^ and 1037 cm^−1^, indicating the cleavage of ester bonds during the reaction and the generation especially of carboxyl groups^50^. These observations provide evidence of the chemical changes occurring during the hydrothermal process, specifically the transformation of ester groups into carboxyl groups. The second hydrothermal cycle, performed in the presence of GnO, induces structural changes in HySB material (Fig. 6), as evidenced by the decrease in the peak at 1695 cm^−1^, the complete disappearance of the peak in the 900–1080 cm^−1^ region and decrease in intensity of the peak from 1084 to 1380 cm^−1^. Simultaneously, the other peaks exhibit changes in position and intensity. These modifications suggest a reduction in the concentration of oxygen-containing functional groups, aligning with the Raman spectroscopy data.

### Electrochemical characterization

The electrochemical behavior of commercial graphite-supercapacitor electrode material, labelled spherical graphite (SG) versus HySB sample was studied both in half-cell and full-cell configurations. A classical electrochemical cell with three electrodes was used for the half-cell configuration while a CR 2016-coin cell system was used for the full-cell configuration. The counter electrode was a platinum plate, while the reference electrode was an Ag/AgCl electrode. The samples had a geometric surface area of 2.3 cm^2^. On the frequency range between 100 kHz and 1 mHz, impedance measurements were performed with an AC wave of about 10 mV (peak-to-peak) superimposed on a DC bias potential, as well as at the open circuit potential of each sample and at the optimum potential extracted from the capacitance (C) versus (potential (E) diagram. The impedance data were obtained at a rate of 10 points per decade change in frequency. Charge–discharge tests were running at a constant current of ± 1 mA in the potential range of 0–1 V. All half-cell tests have been performed in 0.5 M H_2_SO_4_ at room temperature, 1.012 ± 5 hPa under atmospheric oxygen conditions without agitation.

**Figure 7:**
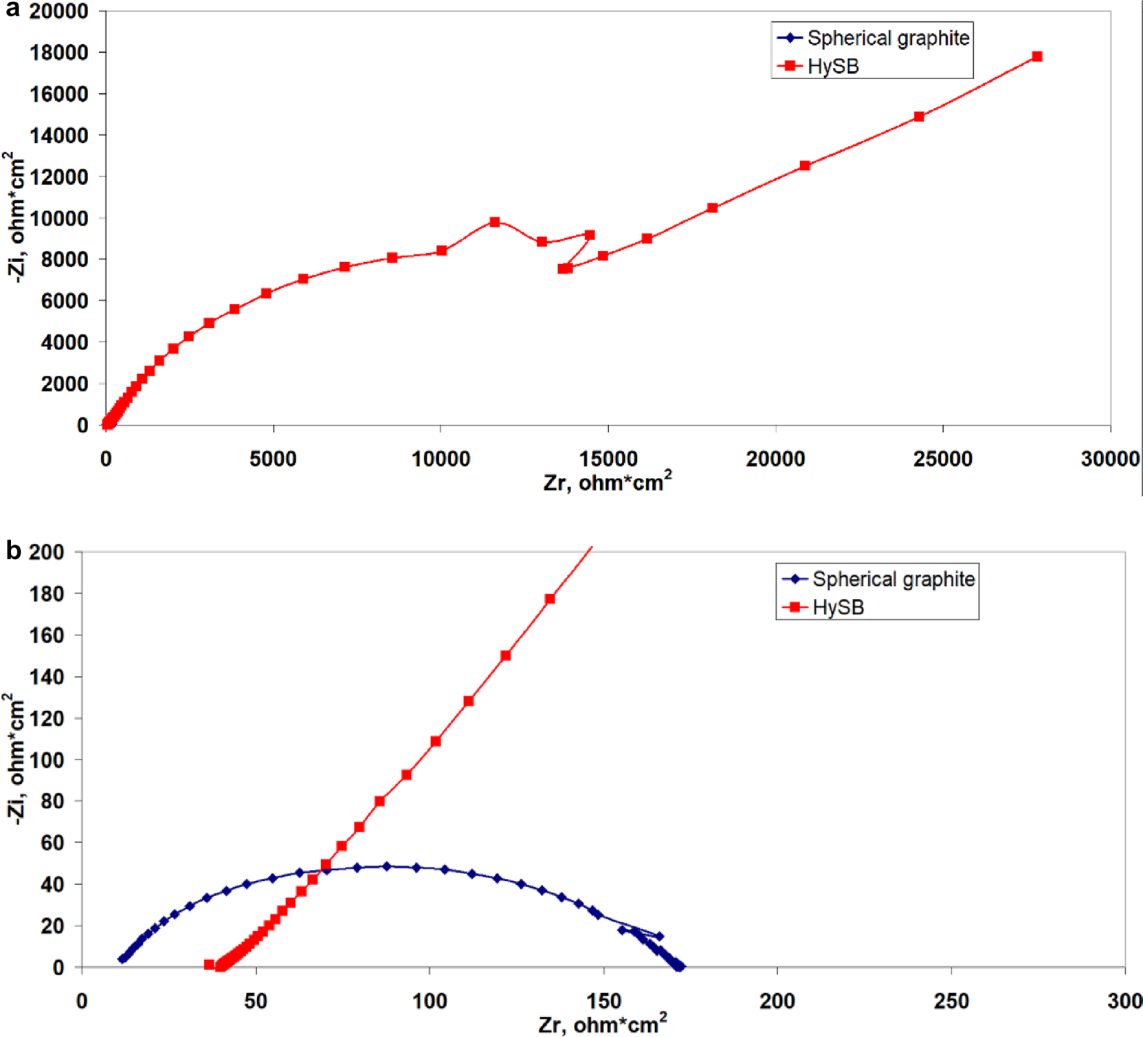
(**a**) Electrochemical impedance spectroscopy: Impedance spectra in Nyquist plots. The figure presents the complex plane (the imaginary component -Zimaginary versus the real component Zreal of impedance) for the spherical graphite (blue lines) and HySB (red lines) samples. (**b**) Nyquist plots (zoomed-in view). This figure is a zoomed-in view of the previous figure (Fig. 7) in order to make it possible to see both curves for the spherical graphite (blue lines) and HySB (red lines).

**Figure 8:**
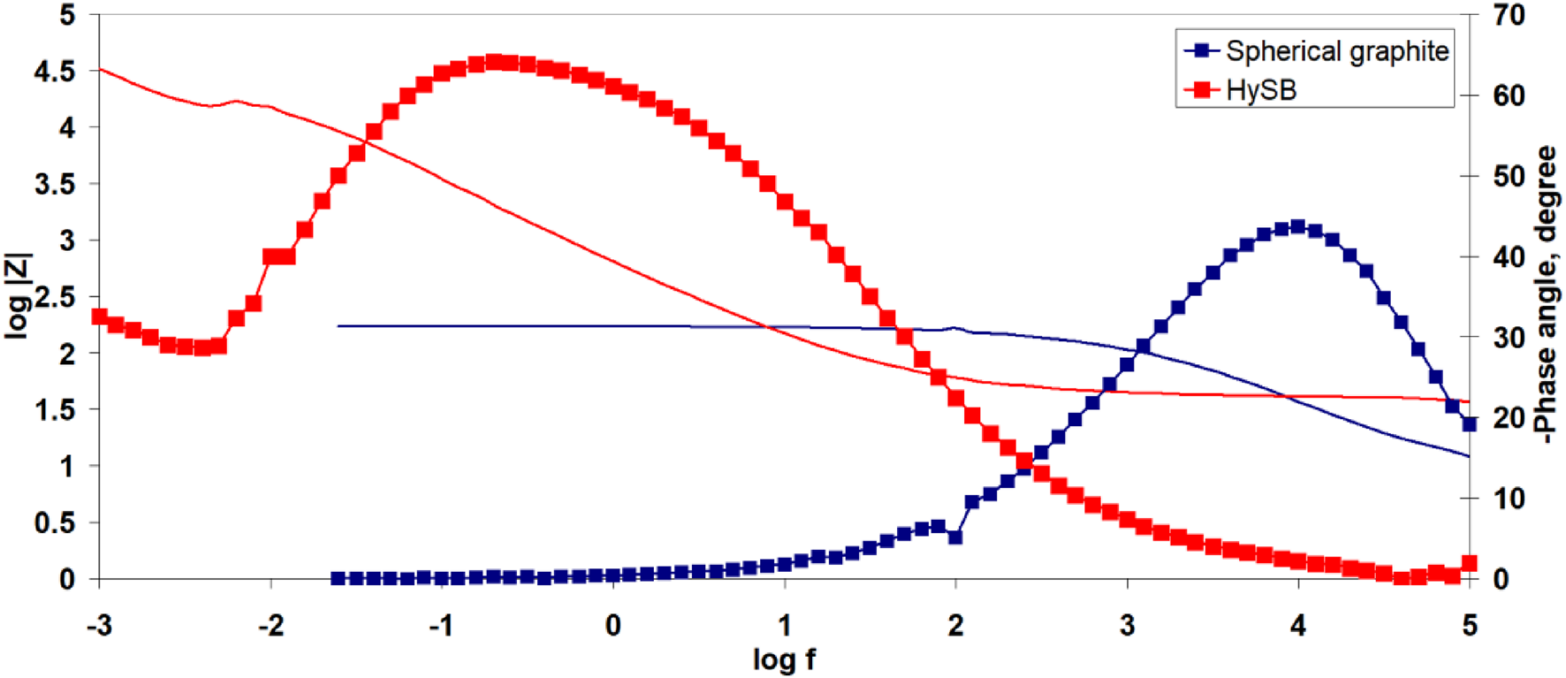
Electrochemical impedance spectroscopy: Bode phase angle plot (dot lines) and Bode magnitude plot (straight lines). The figure presents the Bode diagram (log|Z| vs. log f—straight lines and Phase angle vs. log f—dotted lines) for the spherical graphite (blue lines) and HySB (red lines) samples.

Figure 7a presents the Nyquist diagrams corresponding to the commercial (spherical) graphite labeled SG and HySB samples. Both curves cannot be simultaneously represented in the same complex plane due to significantly higher impedance component values exhibited the HySB sample. In order to visualize the Nyquist diagram corresponding to the commercial graphite sample, Fig. 7b was generated, showing a zoomed-in view of Fig. 7a at low-impedance components values. It can be observed that both samples exhibit a single Debye semicircle, consistent with the appearance of a single time constant in the Bode diagrams (see Fig. 8) and suggesting the presence of a single interface in both samples.

Every point on the Nyquist diagram corresponds to a distinct frequency value. By multiplying the Z′ and Z″ axes with the geometric surface area of the electrode, can be obtained area-specific resistance and impedance values^51^.

Figure 8 illustrates the Bode diagrams for the studied samples. It can be observed that both samples exhibit a single time constant, which appears at high frequencies (10^4^ Hz) for the spherical graphite and at low frequencies (10^–1^ Hz) for the HySB sample. The phase angle corresponding to the time constant in the case of spherical graphite (45°) indicates that the interface processes are diffusion-controlled. On the other hand, for the HySB sample, the phase angle has a value of approximately 65°, indicating moderate capacitive behaviour with diffusion tendencies.

**Figure 9:**
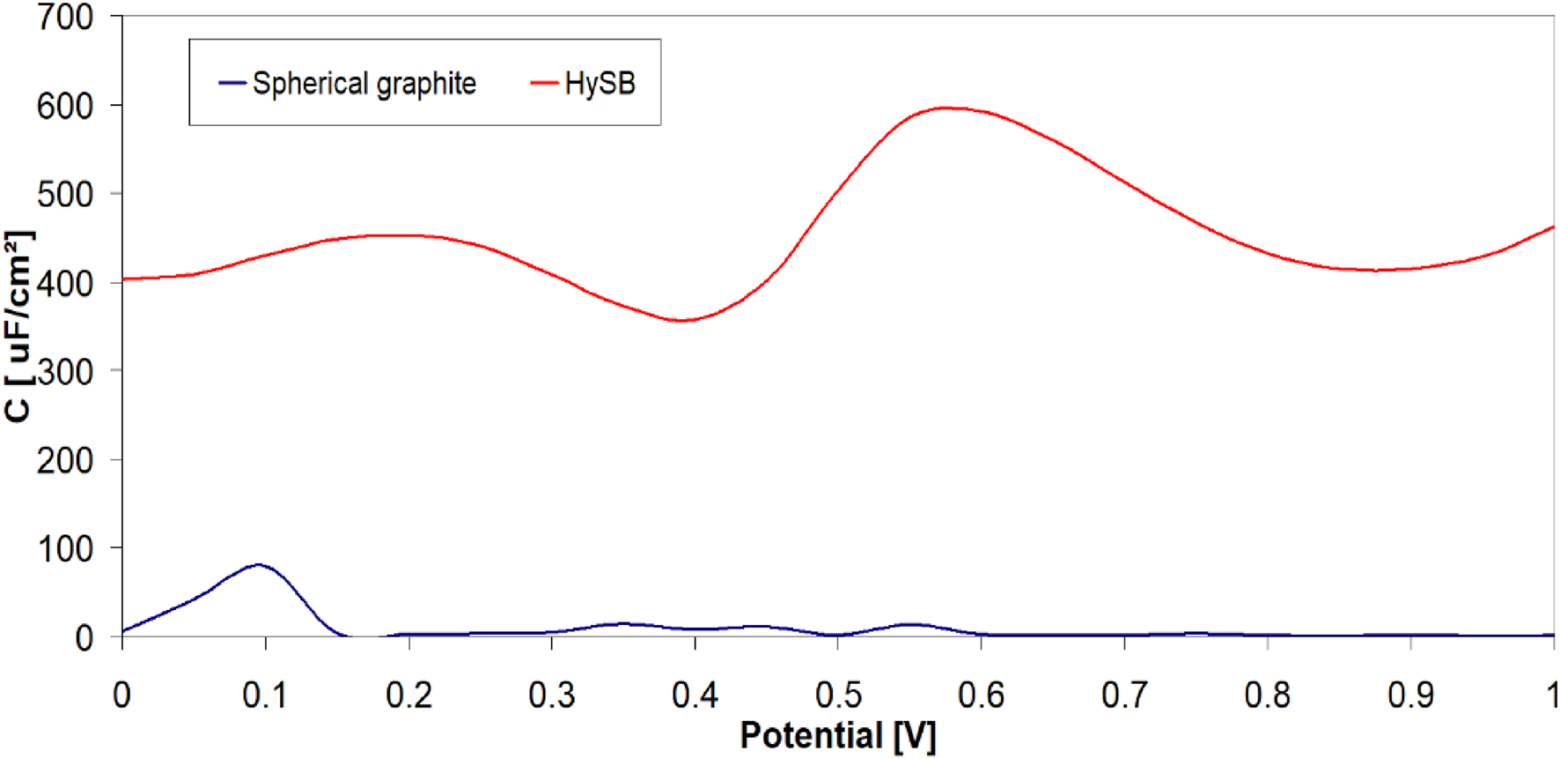
Variation of capacitance (C) with potential (E). The figure presents the variation of determined capacitance with voltage for the spherical graphite (blue line) and HySB (red line) samples. Each capacitance point is determined at a fixed potential value which varies from 0 to 1 V in steps of 50 mV.

The polarization resistance increases by 2 orders of magnitude for the HySB sample at the stationary potential (at open circuit potential, OCP) and by 3 orders of magnitude for the same sample at the optimum potential. This indicates a significant reduction in charge transfer through diffusion in the double-layer, which is confirmed by the increase in double-layer capacitance values (see Table 2). For an ideal capacitor, the expected phase angle is -90°, which is considered optimal for insulating electrodes. This phase angle indicates the maximum possible phase shift between voltage and current. Deviations from this value suggest that the dielectric layer might have a reduced ability to retain an electric charge and reveal electron flow. A phase angle close to − 45° is associated with pseudocapacitance. A reduction in the magnitude of impedance (|Z|) at low frequencies can indicate a decrease in the material’s resistance, leading to enhanced electron flow within the electrode. The upward trend in the imaginary impedance plot indicates the capacitive characteristics of the HySB electrode, covering a broad frequency range. The optimum potential was determined from the variation of capacitance (C) with potential (E) over a range from 0 to 1000 mV (Fig. 9) at the frequencies corresponding to the time constants from the Bode diagrams (Fig. 8).

**Table 2:** Electrochemical Impedance spectroscopy (EIS) parameters.

Sample	Electrolyte	R_el_, kΩ cm^2^	R_p_, kΩ cm^2^	C_dl_, μF cm^−2^
Spherical graphite, at OCP	0.5 M H_2_SO_4_	15.48·10^–3^	161.5·10^–3^	158.3
HySB at OCP	0.5 M H_2_SO_4_	38.09·10^–3^	23.99	530.5
HySB at 600 mV versus Ag/AgCl	0.5 M H_2_SO_4_	3.36·10^–3^	399.5	398.3

**Figure 10:**
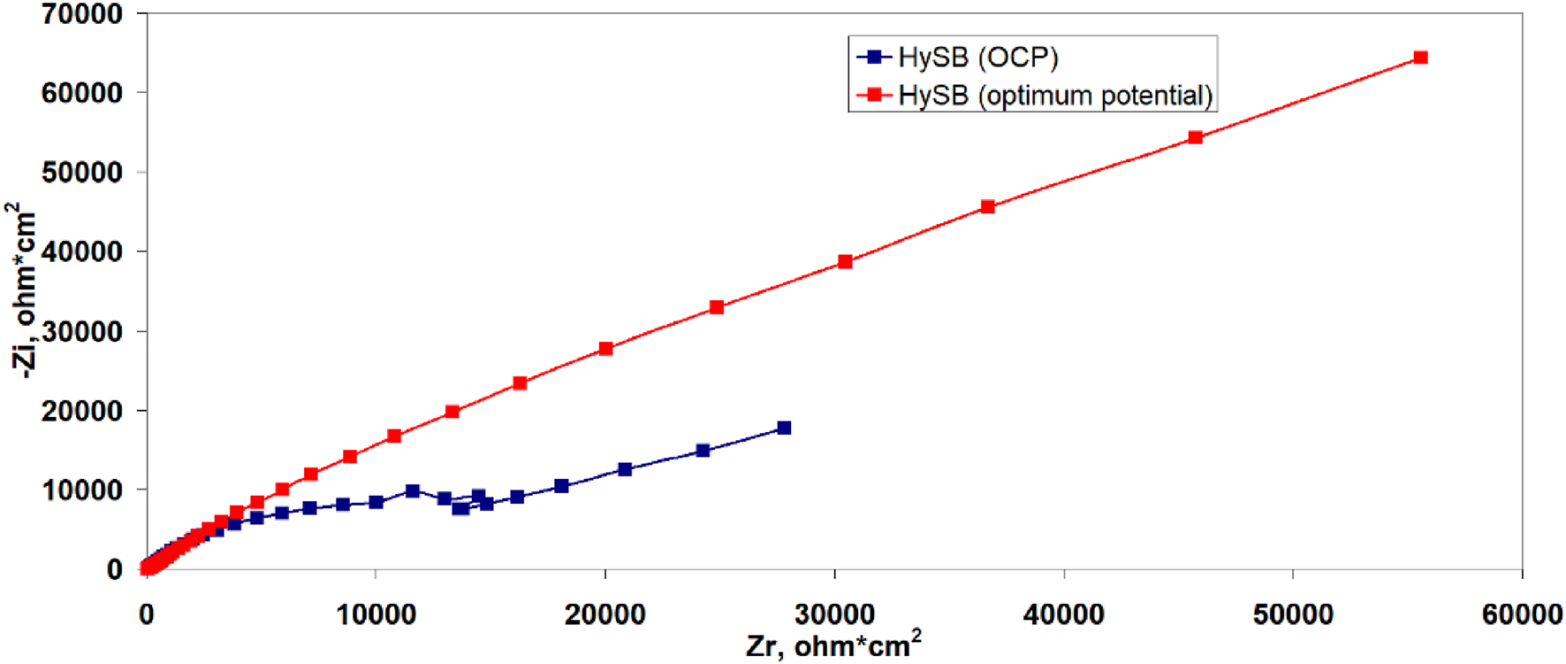
Electrochemical impedance spectroscopy: Nyquist plots for HySB at stationary potential (OCP) and optimum potential. The figure presents the complex plane (the imaginary component –Zimaginary vs. the real component Zreal of impedance) for the HySB sample made at open circuit potential (blue lines) and at optimum potential (red lines). This diagram is necessary for calculating the electrochemical parameters from table no.2.

**Figure 11:**
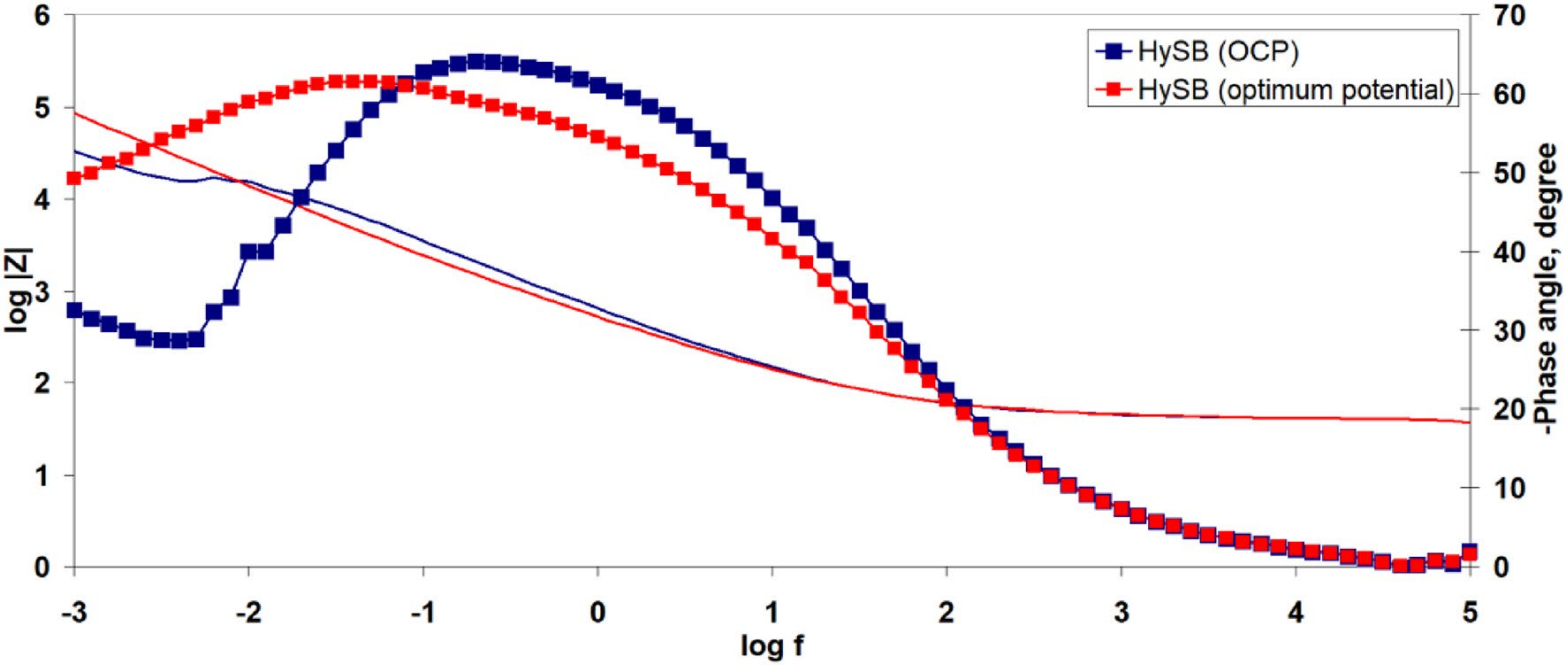
Electrochemical impedance stectroscopy: Bode plots for HySB at stationary potential (OCP) and optimum potential; Bode phase angle plot (dot lines) and Bide magnitude plot (straight line). The figure presents the Bode diagram (log|Z| vs. log f—straight lines and Phase angle vs. log f—doted lines) for the HySB sample at open circuit potential (blue lines) and optimum potential (red lines).

It can be observed that the optimum potential for the HySB sample is reached at approximately 600 mV. At this potential value, the capacitance attains its maximum value, which is consistent with the measurements obtained from electrochemical impedance spectroscopy (see Figs. 10, 11). The significantly superior capacitive behavior of the HySB sample is clearly highlighted in Fig. 9 across the entire potential range. This suggests that the HySB material is a highly attractive candidate for use as a carbonaceous material in the development of electrodes designed for supercapacitors or any other energy storage and conversion device, not only under static conditions (EIS measurements).

The polarization resistance and double-layer capacitance values presented in Table 2 were determined through circular regression with a correlation factor greater than 0.999 from the Nyquist diagrams (see Figs. 7a,b, 10).

**Figure 12:**
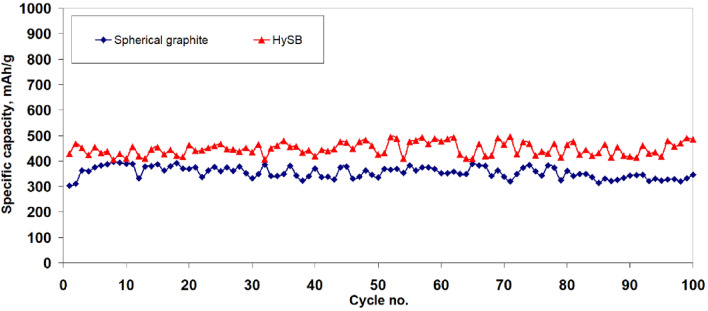
Variation of the specific capacity with no of cycles. The figure presents the variation of specific capacity with cycle number for the spherical graphite (blue line) and HySB (red line) samples. Each specific capacity point is determined from the discharge branch of the charge–discharge cycles.

**Figure 13:**
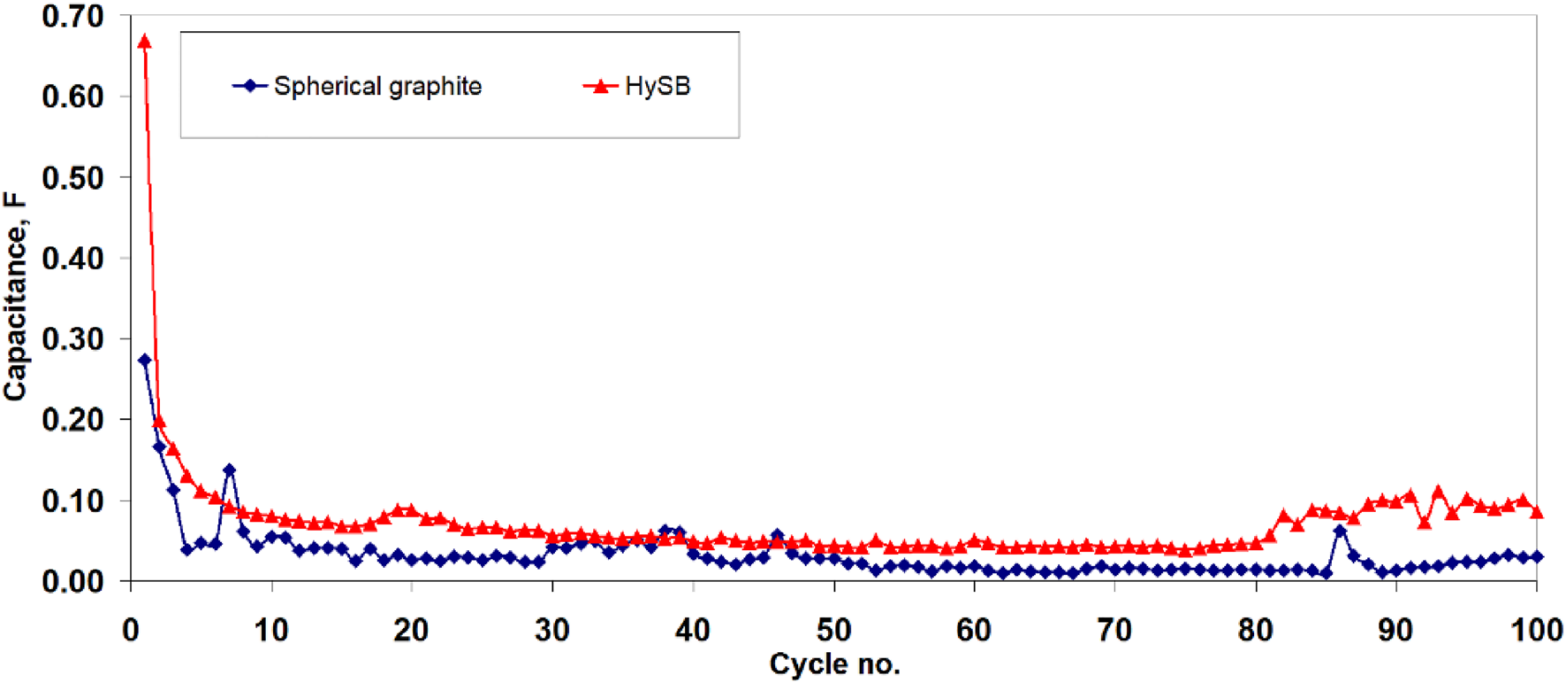
Variation of the specific capacity with no of cycles. The figure presents the variation of capacitance with cycle number for the spherical graphite (blue line) and HySB (red line) samples. Each capacitance point is determined from the discharge branch of the charge–discharge cycles.

Figure 12 illustrates the variation of specific capacity with the number of charge–discharge cycles. It can be observed that for the HySB sample, the specific capacity fluctuates around 450 mAh g^−1^ over the course of 100 cycles, while the spherical graphite exhibits a specific capacity that fluctuates around 350 mAh g^−1^. Both samples show a slight tendency of increasing both specific capacity and capacitance (see Fig. 13) starting from cycle 90, which may indicate improved long-term stability and an increase in electrochemically active surface area through enhanced wettability.

Until now, supercapacitors analysis has primarily depended on the use of impedance spectroscopy and cyclic voltammetry. In addition, some researchers have used computer simulations to perform additional studies^52^. Despite these efforts, no comprehensive model capable of explaining all experimental results has been produced. One important modelling technique is the design of an analogous circuit for a given electrochemical system^53^.

**Figure 14:**
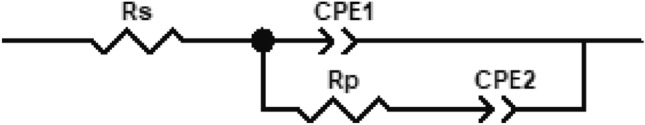
Electrical equivalent circuit for HyBS sample at stationary potential (OCP) used for fitting of EIS measures. This is an electric circuit consisting of a resistor (Rs)—representing the solution resistance—in series with a constant phase element (CPE1), which is in parallel with a series assembly of resistor (Rp)—representing the polarization resistance—and a second constant phase element (CPE2).

**Figure 15:**
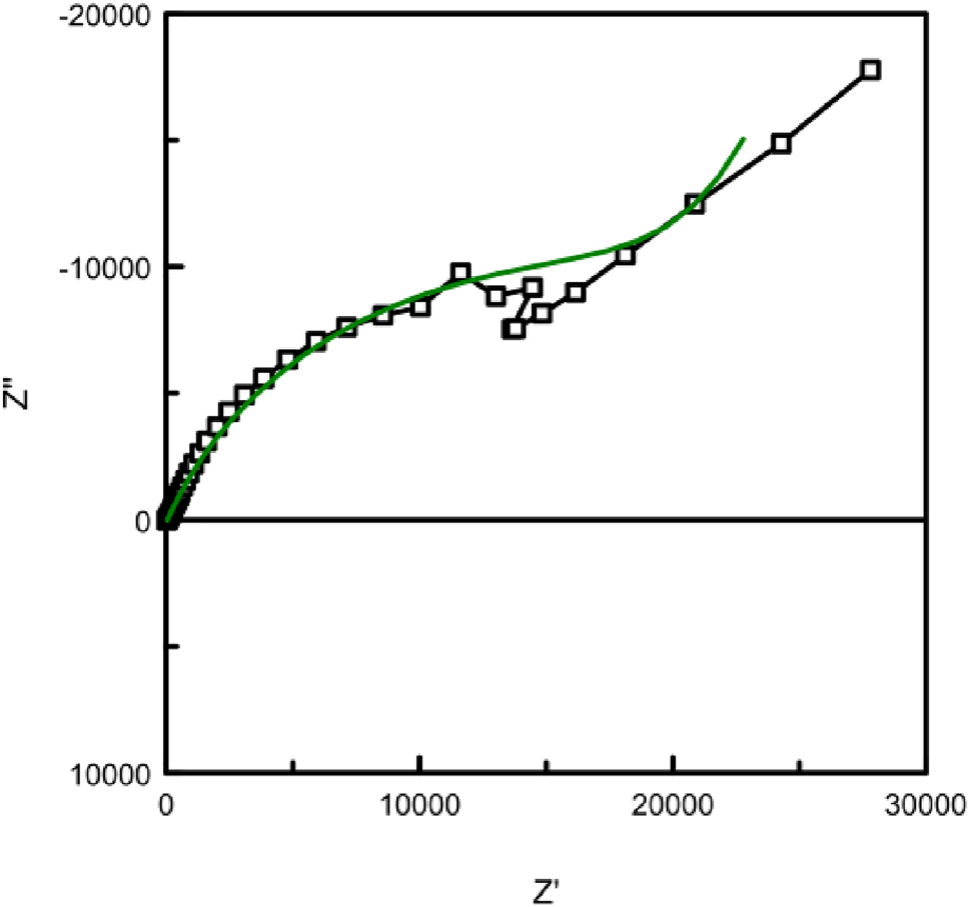
Nyquist diagrams for HyBS sample at stationary potential (OCP) (black-experimental; green-fitted). The figure presents the complex plane (the imaginary component -Zimaginary vs. the real component Zreal of impedance) for the HySB sample made at open circuit potential (dotted line) and fitted results corresponding to equivalent electric circuit presented in Fig. 14 (green straight line).

**Figure 16:**
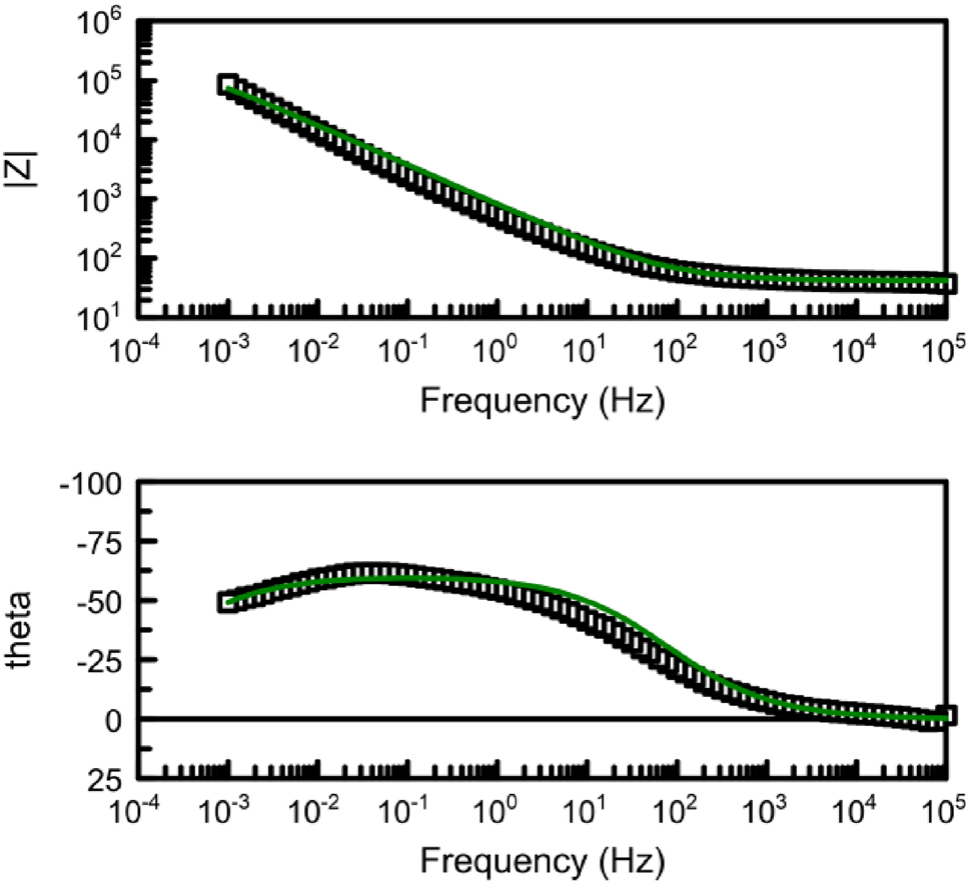
Bode diagrams for HyBS sample at stationary potential (OCP) (black-experimental; green-fitted). The degree of curve fitting in Fig. 16 illustrates the exact correlation between the experimental and theoretical data, which supports the validity of the suggested equivalent circuit.

**Figure 17:**
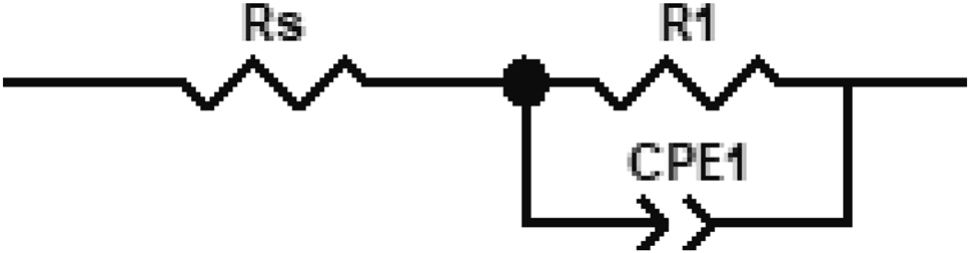
Electrical equivalent circuit for HyBS sample at optimum potential, 600 mV, used for fitting of EIS measurements. This schematic electrical circuit is proposed to simulate the interface between the HySB sample and the electrolyte at the optimum potential. It consists of a resistor RS (representing the solution resistance) in series with a parallel assembly of R1 (polarization resistance) and CPE1 (constant phase element).

**Figure 18:**
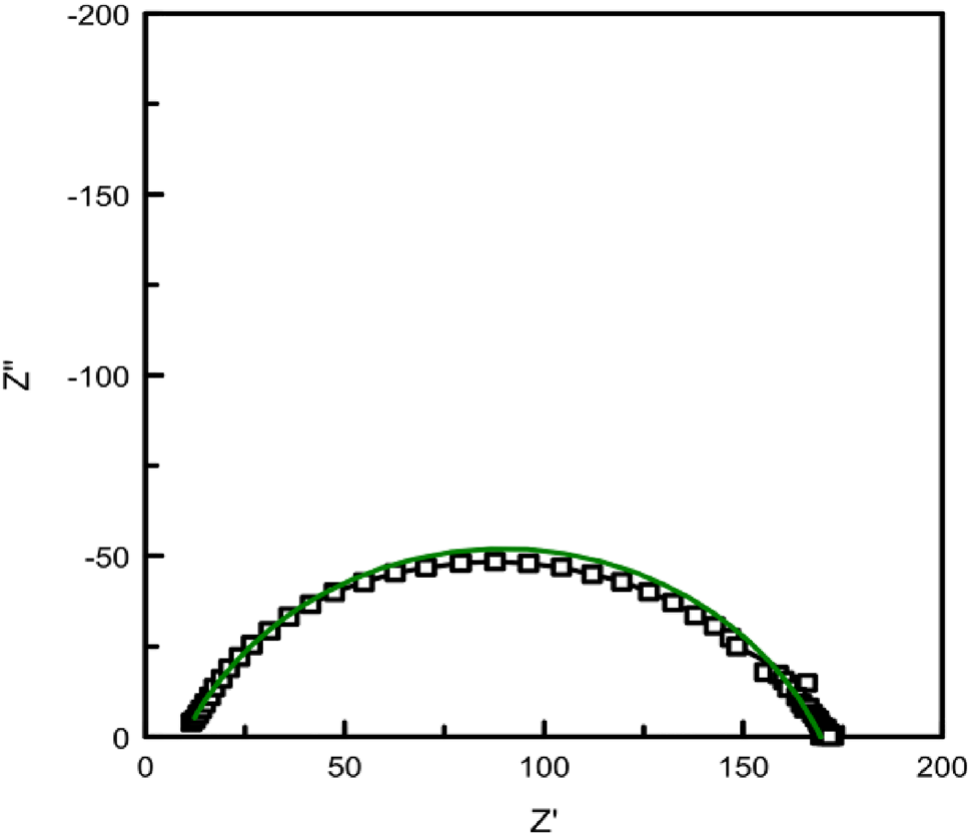
Nyquist diagrams for HyBS sample at 600 mV (optimum potential) (black-experimental; green-fitted). The figure presents the complex plane (the imaginary component -Zimaginary vs. the real component Zreal of impedance) for the HySB sample made at optimum potential (dotted line) and fitted results corresponding to equivalent electric circuit presented in Fig. 17 (green straight line).

**Figure 19:**
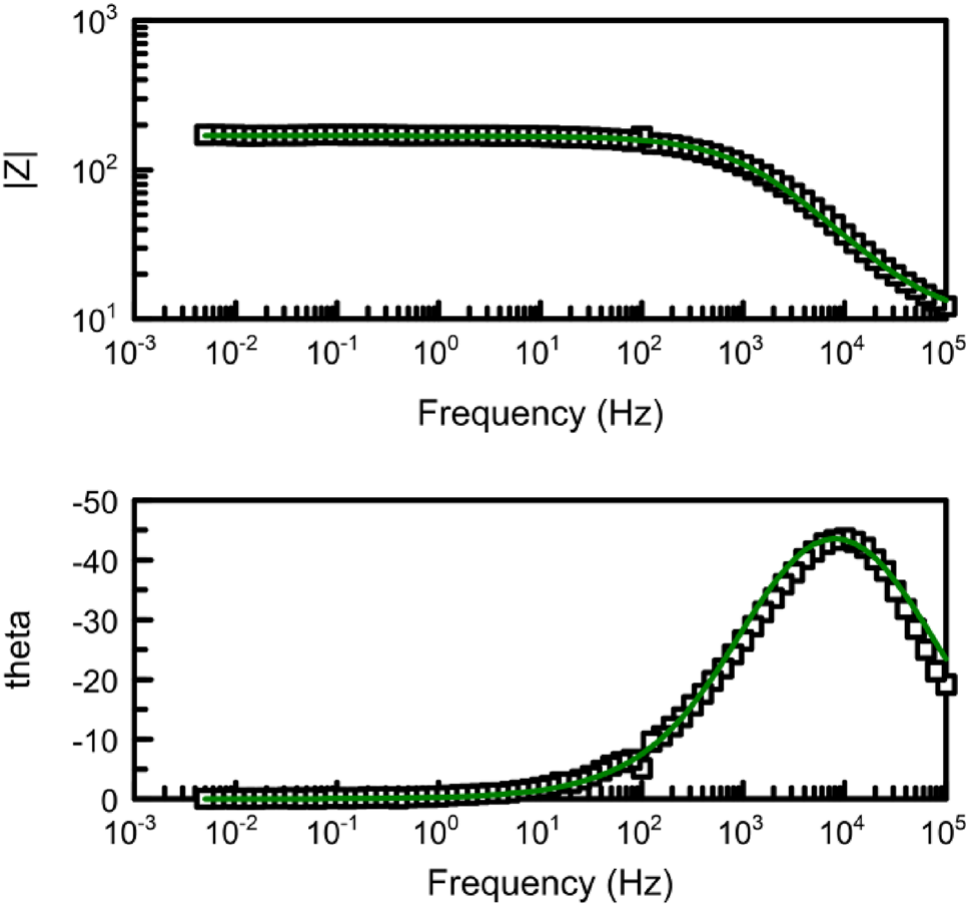
Bode diagrams for HyBS sample at optimum potential, 600 mV (black-experimental; green-fitted). The figure presents the Bode diagram (log|Z| vs. log f and Phase angle vs. log f) for the HySB sample at open circuit potential (dotted line) and fitted results corresponding to the equivalent electric circuit presented in Fig. 17 (green straight line).

In our study, based on the experimental results from electrochemical impedance spectroscopy measurements, equivalent electrical circuits have been generated in the ZView simulation software. The results are presented in Figs. 14, 15, 16, 17, 18 and 19.

The best match between experimental and fitted results is obtained in the case of spherical graphite sample (see Figs. 18, 19) on the whole range of frequency using a simple electrical equivalent circuit (Fig. 17), identical to the one used in the case of HySB sample at optimum potential.

There is a good match between the experimental and simulated curves over a wide frequency range (from 100 kHz to approximately 5 mHz). The proposed equivalent electrical circuits (Figs. 14, 17) for both sets of experimental data (in stationary conditions, OCP, and at optimum potential, 600 mV respectively) largely capture the imagined mechanism for the interface phenomena. The presence of an additional constant phase element in the equivalent circuit of the HySB sample (in series with the polarization resistance), measured under stationary potential, may lead to the hypothesis that the diffusive branch observed at low frequencies in the Nyquist plot **(**Fig. 10) could be a second Debye semicircle, a hypothesis unsupported by the visible appearance at constant time in the Bode plot (Fig. 11). In the case of the same sample measured at 600 mV, the proposed equivalent electrical circuit is a classic one, consisting of a resistance (solution resistance) in series with a capacitor (the constant phase element representing an imperfect capacitor associated with a phase angle less than 90°), connected in parallel with a polarization resistance.

### Contact angle measurement

In order to optimize the specific capacity of supercapacitors through appropriate electrode material design, the wetting degree of the electrode in contact with various aqueous electrolytes, is taken into consideration. Current studies have drawn attention to the importance of the relationship between wettability and a supercapacitor's specific capacity^54^. Therefore, in this work, we attempted to assess the wetting degree through contact angle measurements and evaluate the surface energy of the electrode material. The energy density of a supercapacitor can be improved by increasing the wettability since more electrolyte solution may enter into the electrode pores, consequently, surface area being utilized more effectively. The degree of wettability being one of the critical factors that significantly influence the amount of accumulated charge^55^ the compatibility of the electrode and electrolytes become a crucial consideration in the design of a high-performance supercapacitor. The standard method to determine the surface energy of a solid material relay in measuring of the contact angles of pure liquids (liquids with specific surface tension respectively surface tension parameters on a particular solid surface). Table 3 lists the polar and dispersive surface energy components of the three test liquids used in this study, namely, water, ethylene glycol (99.8%), and glycerol (99%)^56^.

**Table 3:** Surface tension and its dispersive/polar components of wetting liquids^56^.

Liquid	Room temperature Surface tension, mN m^−1^	Dispersive component, mN m^−1^	Polar component, mN m^−1^
Water	72.8	26.4	46.4
Glycerol	63.4	37	26.4
Ethylen glycol	47.7	26.4	21.3

In Table 4 are given the obtained contact angle values for commercial graphite and HySB sample.

**Table 4:** Contact angle values of samples.

Sample	Liquid	Contact angle, °	
HySB	Water	94.609	
	Glycerol	87.144	
	Ethylene glycol	53.037	
Spherical graphite	Water	128.306	
	Glycerol	108.337	
	Ethylene glycol	105.035	

Analysing the registered values provided in Table 4, it is clear that the HySB sample has a significantly superior wetting characteristics given by decreased contact angle value compared with the commercial material used as reference. The Surface energy and dispersive /polar components of samples have been calculated based on Owens/Wendt’s two-component model for solid surface energy^56^. The surface free energy (σ_S_) of a solid is defined as the change of the total surface free energy (*G*) per surface area (*A*) at constant temperature (*T*), pressure (*P*) and moles (*n*)^52–57^:

1 $$\sigma_{S} = (\delta G/\delta A)_{T,P,n}$$

Measurement of the contact angles created by test liquids with known surface tensions, including both their dispersive and polar components, can be used to determine the surface energy of a solid material. Using the right model, these elements are used to determine the interfacial tension between the solid and the liquid. The Owens, Wendt, Rabel, and Kaelble (OWRK) model is a frequently employed one that accounts for both the solid's surface energy as well as the geometric average of the dispersive and polar components of the liquid's surface tension^53^. Owens and Wendt combined the equations of Good and Young to produce the following equations^57,58^:

2 $$\sigma_{SL} = \sigma_{S} + \sigma_{L} - 2(\sigma_{L}^{D} \sigma_{S}^{D} )^{1/2} - 2(\sigma_{L}^{p} \sigma_{S}^{P} )^{1/2}$$

wherein: σ_L_ = overall surface tension of the wetting liquid, σ_L_^D^ = dispersive component of the surface tension of the wetting liquid, σ_L_^P^ = polar component of the surface tension of the wetting liquid, σ_S_ = overall surface energy of the solid, σ_S_^D^ = dispersive component of the surface energy of the solid, σ_S_^P^ = polar component of the surface energy of the solid, σ_SL_ = the interfacial tension between the solid and the liquid, and θ = the contact angle between the liquid and the solid.

Substituting Eq. (2) in the Young equation

3 $$\sigma_{S} = \sigma_{SL} + \sigma_{L} \cos \theta$$

can be obtained:

4 $$\frac{{\sigma_{L} (\cos \theta + 1)}}{{2(\sigma_{L}^{D} )^{1/2} }} = (\sigma_{S}^{P} )^{1/2} \frac{{(\sigma_{L}^{P} )^{1/2} }}{{(\sigma_{L}^{D} )^{1/2} }} + (\sigma_{S}^{D} )^{1/2}$$

In graphical representation:

5 $$Y = \frac{{\sigma_{L} (\cos \theta + 1)}}{{2(\sigma_{L}^{D} )^{1/2} }};\;X = \frac{{(\sigma_{L}^{P} )^{1/2} }}{{(\sigma_{L}^{D} )^{1/2} }}$$

**Figure 20:**
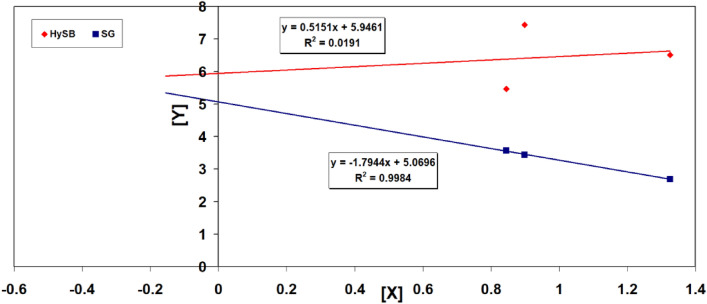
Representation of Owens/Wendt equation for HySB and commercial graphite (SG) samples.

The measured contact angle as well as the dispersive and polar components of the test liquid's surface tension are represented by the variables y and x in this equation. The dispersive and polar components of the solid's surface energy, which are being sought, are represented by the regression line's axis intercept (c) and the slope (m). By measuring the contact angles of at least two test liquids, these parameters can be determined through the evaluation of the regression line. In Fig. 20 is given the representation of Owen/Wendt equation for HySB and commercial graphite, SG, and in Table 3 dispersive and polar components of samples, calculated with the Owens/Wendt two-component model for solid surface energy.

A surface with high wettability possesses a surface energy that generates a strong attractive force, resulting in the liquid droplet spreading out on a surface rather than forming a distinct droplet shape. The wettability is enhanced when both the surface energy of the solid substrate and the surface tension of the liquid are higher, leading to a smaller contact angle as in HySB sample.

## Conclusions

In this study, a new hybrid graphene oxide-bio-based char as electrode material for supercapacitors was developed. The electrode material was synthesized through a low-temperature HTC process, which is a more energy-efficient approach for transforming lignocellulosic wastes into carbonaceous materials.

The new material was compared in terms of supercapacitors functional characteristics with a commercially available graphite, commonly used in these applications. This study proved that properties such as wettability, surface energy and double-layer capacitance are significantly superior to those of commercial material. The polarization resistance of the HySB sample increases by a factor of 100 at the stationary potential (open circuit potential, OCP), and by a factor of 1000 at the optimal potential. This suggests a substantial decrease in charge transfer via diffusion in the double-layer, which is supported by the observed rise in double-layer capacitance values.

Additionally, two equivalent electrical circuits have been suggested using electrochemical impedance spectroscopy measurements. The comparison of experimental and simulated curves at different frequencies shows a strong correlation, indicating that the proposed equivalent electrical circuits effectively depict the underlying mechanism of interface phenomena in both sets of experimental data.

HySB’s superior performance is not limited to static conditions, as demonstrated by the EIS measurements.

All the experimental data suggest that bio-based micro-nanostructures obtained through a sequential and cost-efficient path involving a minimal functionalization can successfully replace certain existing commercial materials used in the relevant market of energy storage devices.

## Materials and methods

### Chemicals and reagents

Throughout the study, several chemicals and reactive agents of analytical purity (provided by Sigma-Aldrich) were used: zinc chloride (ZnCl_2_), sulphuric acid (H_2_SO_4_ 98%), nitric acid (HNO_3_ 38%), hydrochloric acid (HCl 0.1 M), Fenton reagent, N-methyl-2-pyrrolidone and polyvinylpyrrolidone, natural graphite 10 mesh, ethylene glycol (99.8%), and glycerol (99%), Toray carbon paper. Commercial graphite: SLC1512P (19 µm) used as reference supercapacitor electrode material during measurements was provided by Superior Graphite, USA.

### Bio-based char synthesis

The spruce bark (SB), used as biomass precursor, was provided as waste for forestry and wood processing industry. Prior to use, the spruce bark was air-dried at room temperature and grounded in a laboratory mill until achieving particles size ranging from 0.1 to 2 mm. Next, a homogenous paste was obtained by combining 200.0 g of spruce bark with ZnCl_2_ at a weighted ratio of 1:0.5 and 400 mL of distilled water. The paste was then dried in a vacuum oven at 105 °C for 10 h. In order to convert the biomass into a char, a hydrothermal carbonization (HTC) process was applied. Thus, 100 g of activated SB was dispersed in 600 ml distilled water in a stainless-steel autoclave. Throughout 5 h the reactor was enclosed and heated to 200 °C. The stainless-steel autoclave was allowed to drop down to ambient temperature after HTC procedure, giving enough time for depressurization. To separate the solid (hydrochar, HC) from the liquid phase, the reaction mase was filtered. Distilled water was then used to wash the solid phase. After that, the HC was dried at 105 °C until a constant mass was attained, weighed and kept for later characterization. This sample was labelled SB1.

#### Functionalisation of bio-based char

##### Synthesis of graphite oxide

For the synthesis of graphite oxide, the Hummers method^59–67^ was employed with the following modifications: 45 ml of sulphuric acid (H_2_SO_4_) and 9 ml of nitric acid (HNO_3_) (volume ratio 3:1) were mixed and stirred for several minutes. Then, 1 g of natural graphite flakes was added to the solution while keeping the solution at 50 °C under stirring conditions. Next, 10 ml of Fenton’s reagent was added slowly into the mixture with continuous stirring for 5 h. The obtained dispersion of graphite oxide was washed with 0.1 M HCl and then with distilled water several times until a pH of 6.5 was reached. The graphite oxide was dried under vacuum for 8 h at 60 °C. The specimen was identified as GO.

##### Synthesis of graphene oxide solution

The graphene oxide was prepared by exfoliation through mild sonication of the graphite oxide by using a simple ultrasonic bath. Thus, 2 mg of GO was dissolved in 200 ml of deionized water under ultrasonication for 2 h at 60 °C. Obtained homogenous dispersion was designated as GnO.

##### Synthesis of GnO-biochar hybrid material

A second run of a hydrothermal carbonization (HTC) process was applied. Thus, 50 g of SB1 was dispersed in 100 ml of GnO solution and placed into the same autoclave at 230 °C for 12 h. The solid phase was filtered and washed with distilled water and dried at 60 °C for 8 h. The material was marked as HySB.

##### Electrode preparation

In this stage, 0.05 g of HySB, is mixed with a binder composed of N-methyl-2-pyrrolidone and polyvinylpyrrolidone in a volumetric ratio of 1:0.5. The resulting homogeneous solution is dripped onto a disc-shaped carbon paper support with a diameter of 1 cm. The sample was dried in a vacuum oven at 80 °C for 5 h. Thus, an electrode with a mass loading of less than 6 mg cm^−2^ of the active compound (HySB) was obtained. The hybrid activated spruce bark-graphene oxide electrode manufactured in this manner will be subjected to further electrochemical evaluations in order to point out its specific functional performances as required for an SC electrode.

##### Characterization methods

The morphology of the samples was studied by scanning electron microscopy (SEM) using a Carl Zeiss SMT FESEM-FIB (Scanning Microscope Tunneling Field Emission Scanning Electron Microscope-Focused Ion Beam) Auriga, Oberkochen, Germany. SEM studies were performed on uncovered samples fixed on platinum-coated supports.

The Raman spectroscopy was conducted using a LabRAM HR Evolution Horiba dispersive Raman spectrometer (Horiba Jobin–Yvon, Palaiseau, FR). A laser with an excitation wavelength of 532 nm and a power of 5 mW was used to prevent sample heating. Raman spectra were collected over the spectral range of 100–3400 cm^−1^, with an acquisition time of 10 s and 10 accumulations. The spectrometer employed a diffraction grating with 1,800 grooves mm^−1^ and a 50 × objective lens with a numerical aperture of 0.75.

The FTIR analysis was conducted utilizing a JASCO FTIR-4200 spectrometer (Jasco.Inc, Tokyo, JP) in transmission mode. The covered spectral range was 450–4000 cm^−1^, with 100 accumulations per spectrum and a resolution of 4 cm^−1^. The KBr pellet method (0.1% w/w) was employed for the analysis.

For electrochemical measurements, a single-channel potentiostat/galvanostat VoltaLab 40 with dynamic EIS (Electrochemical Impedance Spectroscopy) connected to a computer through VoltaMaster 4 Software interface and a multi-channel Netware battery tester was used for the electrochemical characterization.

Contact angle measurements were conducted with a lab-made device composed of a XYZ mobile table and a DinoLite microscope at ambient temperature (22 ± 1 °C) and relative humidity (20–40%). The contact angle was calculated by DinoLite software.

## Data Availability

All data generated or analysed during this study are included in this published article.
